# Maternal Immune Activation and Interleukin 17A in the Pathogenesis of Autistic Spectrum Disorder and Why It Matters in the COVID-19 Era

**DOI:** 10.3389/fpsyt.2022.823096

**Published:** 2022-02-17

**Authors:** Michael Carter, Sophie Casey, Gerard W. O'Keeffe, Louise Gibson, Louise Gallagher, Deirdre M. Murray

**Affiliations:** ^1^INFANT Research Centre, University College Cork, Cork, Ireland; ^2^Department of Paediatrics and Child Health, University College Cork, Cork, Ireland; ^3^National Children's Research Centre, Dublin, Ireland; ^4^Department of Anatomy and Neuroscience, University College Cork, Cork, Ireland; ^5^Department of Psychiatry, School of Medicine, Trinity College Dublin, Dublin, Ireland; ^6^Trinity Translational Medicine Institute, St. James's Hospital, Dublin, Ireland

**Keywords:** ASD, autism, cytokine, maternal immune activation, MIA, interleukin-17A (IL-17A), COVID-19

## Abstract

Autism spectrum disorder (ASD) is the commonest neurodevelopmental disability. It is a highly complex disorder with an increasing prevalence and an unclear etiology. Consensus indicates that ASD arises as a genetically modulated, and environmentally influenced condition. Although pathogenic rare genetic variants are detected in around 20% of cases of ASD, no single factor is responsible for the vast majority of ASD cases or that explains their characteristic clinical heterogeneity. However, a growing body of evidence suggests that ASD susceptibility involves an interplay between genetic factors and environmental exposures. One such environmental exposure which has received significant attention in this regard is maternal immune activation (MIA) resulting from bacterial or viral infection during pregnancy. Reproducible rodent models of ASD are well-established whereby induction of MIA in pregnant dams, leads to offspring displaying neuroanatomical, functional, and behavioral changes analogous to those seen in ASD. Blockade of specific inflammatory cytokines such as interleukin-17A during gestation remediates many of these observed behavioral effects, suggesting a causative or contributory role. Here, we review the growing body of animal and human-based evidence indicating that interleukin-17A may mediate the observed effects of MIA on neurodevelopmental outcomes in the offspring. This is particularly important given the current corona virus disease-2019 (COVID-19) pandemic as severe acute respiratory syndrome coronavirus 2 (SARS-CoV-2) infection during pregnancy is a potent stimulator of the maternal immune response, however the long-term effects of maternal SARS-CoV-2 infection on neurodevelopmental outcomes is unclear. This underscores the importance of monitoring neurodevelopmental outcomes in children exposed to SARS-CoV-2-induced MIA during gestation.

## Introduction

Autism spectrum disorder (ASD) is a neurodevelopmental disorder characterized by a spectrum of deficits in social interactions and communication combined with stereotypical and repetitive behaviors. Up to 50% of those affected can have intellectual disability (ID) and limited verbal communication (1–3). In recent decades, the prevalence of ASD has consistently increased from approximately 1 in 1,000 in the 1960s (4), to 1 in 44 today in the United States (5). Increasing prevalence may in part, be explained by changes in reporting practices, increased recognition of ASD symptoms, broadening of the ASD diagnosis (1), and improved accessibility to services (6, 7). A significant ratio of 4:1 from male to female still exists with markedly differing prevalence rates between the sexes, 1/38 in males and 1/151 among females (8). Although genetic susceptibilities are recognized, the mechanism of disease development is unknown and does not follow a clear pattern of inheritance (9, 10). This suggests possible mediation by additional unknown biological or environmental factors (11). Both common and rare genetic risk factors have been identified with more than 400 diverse genes now linked to ASD. Singly, these genetic factors each convey only a modest increase in ASD risk (~1%), however collectively they can contribute to a far greater risk (12, 13). Up to 20% of individuals with ASD may possess copy number variants (CNVs) and *de novo* loss of function single nucleotide variants (SNVs) that are individually rare but in combination, increase an individual's ASD risk (12). While newer methods of genetic analysis (such as whole genome sequencing) are uncovering new candidate genes with regularity (14), the heterogeneity of the clinical and phenotypic groups within ASD strongly suggest that in those with a genetic predisposition, environmental factors may act in concert to bring about a multisystem dysfunction leading to ASD. A well-characterized environmental factor known to impact early fetal brain development and increase ASD risk is maternal inflammation during pregnancy, which is commonly called maternal immune activation (MIA). Numerous epidemiological studies have linked gestational infections with elevated risk of ASD in offspring (15–17), and animal models of MIA have simulated gestational infection resulting in MIA-induced neural and behavioral abnormalities analogous to those seen in ASD (18–20).

Focused early intervention in young children with ASD has been shown to result in normalized patterns of brain activity, and is associated with improved functional outcomes and reduced morbidity (21, 22). Most children affected by ASD can have a reliable and stable ASD diagnosis from as early as 14 months of age (23), yet in spite of this, the average age of ASD diagnosis is closer to 5 years (24, 25). Numerous studies sought to identify blood-based biomarkers of ASD in affected adolescents and adults (26, 27) and have reported alterations of molecules involved in iron transport (28), inflammation (29, 30), brain development (31), and metabolism (32). None to date has identified and validated reliable mechanistic biomarkers with the ability to improve ASD detection in the crucial early developmental period. Multiple descriptive ASD biomarkers such as characteristic MRI brain findings, abnormalities of gaze preference on eye tracking or characteristic EEG findings in infants with ASD; show promise in terms of aiding earlier ASD detection. However, none is directly involved in the pathogenesis of ASD and arises of the condition rather than contributes to it. The infant brain doubles in volume over the first year coinciding with maximal neuroplasticity and synaptogenesis. Recognition of an early mechanistic biomarker gives us the best chance of implementing strategies during this critical early childhood window allowing ASD diagnosis and intervention at the earliest possible stage.

Here, we highlight recent research in this area, both from pre-clinical animal studies and epidemiological human studies, along with a proposed mechanistic pathway, that we can encourage other research groups with access to suitable maternal-child cohorts to examine this question. We encourage researchers to look at the prospective study of children born during the corona virus disease-2019 (COVID-19) era, when their gestations may have been complicated by mild or even asymptomatic severe acute respiratory syndrome coronavirus 2 (SARS-CoV-2) infection. Otherwise, the long-term effect, if any, of COVID-19 on the fetal brain could remain unknown for years to come.

## Inflammation, Viral Infection, and ASD: What Are the Implications of the COVID-19 Pandemic?

There is growing scientific evidence that aberrant immune activation occurs in ASD (27, 33) based on studies of autistic children and young adults (34, 35). As early as 1971, Stella Chess reported ASD cases associated with the 1964 Rubella outbreak in the United States (36), and in a 1977 follow up study, Chess et al. quoted ASD prevalence rates of 8–13% in children of mothers who were infected during that outbreak (16). Large epidemiological studies indicate that conditions such as maternal autoimmune disorders and mid-trimester viral infections that trigger gestational pro-inflammatory states (i.e., MIA), are linked with elevated ASD, schizophrenia, and bipolar disorder risk in offspring (16, 17, 37, 38). More recently, a range of conditions associated with proinflammatory states in pregnancy such as obesity, psychosocial stress, and pre-eclampsia were associated with increased ASD risk in children (39, 40). Thus, gestational MIA appears to play a role in the pathogenesis of the ASD phenotype in exposed offspring.

## Maternal Immune Activation and Neurodevelopmental Outcomes

We define MIA as a triggering of the maternal immune system by infectious or infectious-like stimuli resulting in an increase in measurable inflammatory markers during pregnancy (41, 42). Maternal immune activation has been most commonly simulated in preclinical rodent, murine and non-human primate (rhesus macaque) animal models by Poly (I:C) (polyinosinic-polycytidylic acid) or LPS (lipopolysaccharide) injection which, respectively, model viral and bacterial infection (18, 43, 44). Poly (I:C) is a synthetic analog of double stranded RNA, mimics the effects of viral infection (45). The triggered immune response results in offspring with behavioral, immunological, and neurological abnormalities that approximate to autistic symptoms observed in humans, notably, impaired sociability and repetitive behaviors (18, 46, 47). Offspring born to poly (I:C) treated dams have consistently, across all exposure categories [administration of varying doses of poly (I:C) and at varying gestations], shown impairment of social interaction, this is manifest as reduced communication in ultrasonic vocalizations (USV) which are usually triggered by separation from the dam in the first two postnatal weeks. Marble burying, a well-recognized behavioral paradigm to measure repetitive behaviors in rodents, again is consistently increased in murine offspring following poly (I:C) treatment (48). These offspring have proven useful in pre-clinical etiological studies as well as identification of therapeutic targets.

Cytokine dysregulation may play a causative role in observed neuronal dysfunction in pre-clinical models of MIA (20, 46, 49). In a recent study, Choi et al. convincingly demonstrated that simulated MIA in murine models leads to elevation in maternal IL-6, which in turn activates maternal Th17 cells. These maternal Th17 cells produce IL-17, which is thought to cross the placenta triggering increased expression of IL-17AR in the fetal brain and leading to cortical malformations and behavioral abnormalities (18, 50). These malformations parallel abnormalities found in brain development in children, adolescents and adults with ASD (51, 52). Poly (I:C) treatment also leads to raised IL-17A mRNA levels in placental tissue of these mice (18). Through inhibition of IL-6 and IL-17A signaling with antibody blockade of the IL-17A cytokine, Choi at al also determined that a sustained increase in IL-17A expression seemed to be pathogenic in ASD, as IL-17A blockade prevented the development of ASD-like phenotypes (18). Specific behaviors in mice which model core diagnostic features of ASD (including repetitive burying and increased neonatal USV) were normalized in the previously MIA-exposed offspring (53, 54).

Improved fetal resilience is associated with lower intensity of MIA. Autism spectrum disorder risk after prenatal exposure to maternal fever has been found to increase in a dose dependent manner (55, 56) and similar effects were identified in animal models of MIA (57). A balanced maternal diet seems to contribute to improved fetal resilience also (58–60). Exposure to relatively higher grades of immune activation *via* high intensity MIA (40), intrapartum infection (61, 62) and genetic risk factors lead to reduced fetal resilience, and increased likelihood of unfavorable developmental outcomes.

## Alterations in Cytokine Expression in Human Studies

While many studies have examined the cytokine profiles of individuals with ASD, only a very limited number of studies to date have examined mid-gestation cytokine levels in mothers of children who subsequently develop ASD. Three studies retrospectively analyzed maternal blood sampled during pregnancy. A 2017 study by Jones et al., reported elevated mid-gestation cytokines and chemokines in mothers of children with ASD associated with ID, and particularly early onset ASD (as defined by the authors as early or sustained delays in language or social skills, and excluding those showing clear skill regression) (63). Dysregulation was noted in a number of cytokines including interleukins IL-1α, IL-1β, IL-2, IL-4, IL-6, IL-8, and IL-17A between 15 and 19 weeks' gestation. An earlier study noted elevations in mid-gestation serum IL-4, IL-5, and IFN-gamma levels in mothers of ASD affected children (15). While, more recently, Irwin et al. demonstrated alterations in IL-4, MCP-1, and IL-10 levels in 28-week gestation serum of mothers who birthed ASD affected children (64). Other authors have examined amniotic fluid at mid-gestation and found elevated levels of IL-4, IL-10, TNF-α, and TNF-β in ASD patients vs. controls (65). Yet, amniotic fluid cytokine concentrations are more indicative of the fetal immune state rather than the maternal state (66, 67). In Table 1, we outline a number of the cytokines most frequently found to be dysregulated in the serum or cerebrospinal fluid (CSF) of ASD affected individuals, and gestational serum and amniotic fluid samples from mothers of ASD affected children.

**Table 1:** Cytokine dysregulation in ASD affected individuals and in gestational serum and amniotic fluid samples of mothers with ASD affected offspring.

**Cytokine**	**Category**	**Altered in blood/CSF of ASD individual**	**Altered in gestational blood**	**Altered in amniotic fluid**	**Cytokine characteristics relevance to ASD**
TNFα	Pro-inflammatory	(29, 68–70)	(63)	(65)	Apoptosis of infected cells. Elevated in the CSF and blood of ASD affected individuals (29, 68, 69).
IL-1β	Pro-inflammatory	(29, 68, 71, 72)	(63)		A potent pro-inflammatory cytokine involved in both acute and chronic inflammation. Correlated with ASD symptom severity (34).
IL-6	Pro-inflammatory	(29, 68, 70–74)	(63)		Induces production of acute phase proteins and stimulates B-cell antibody production (75). Pleiotropic (affects hematologic, hepatic, endocrine, and metabolic function). Thought to impact synapse formation and neuronal migration (76). Potentially mediates IL-17 linked ASD risk in pregnancy (18, 46).
IFNγ	Pro-inflammatory	(27, 29, 73)	(15, 63)		Interfaces between innate and adaptive immune response. Secreted by NK cells, and promotes NK killing. Activates macrophages, which produce IL-12 and−23, stimulating Th1 and Th17 cell, respectively. Inhibits Th2 cells. Versatile, with a role in defense against intracellular pathogens, tumors surveillance, autoimmunity, allergy, and the protection of the amniotic space during pregnancy (77).
IL-17	Pro-inflammatory, Chemotactic	(29, 35, 70, 74, 78, 79)	(63)		Derived from Th17 cells, a subset of CD4 cells. Potentiates the innate PMN response throughout inflammation. Postulated to trigger alterations in the blood brain barrier and lead to cortical dysplasia (46).
IL-4	Pro-/Anti-inflammatory, Allergy	(72)	(15, 63, 64)	(65)	A Th2 derived cytokine, often linked with asthma and allergic type inflammation (33). Dual role: pro/anti-inflammatory properties. Crucially important in mitigating inflammation during pregnancy (primarily through suppression of Th1 T-cells and associated cytokines (IL-2 and IFNγ).
GM-CSF	Growth factor	(80)	(63)		A colony-stimulating factor. Produced by stromal cells, it targets bone marrow, and precursor cells, mediating hematopoiesis.
IL-8	Chemotactic	(71, 73, 81)	(63)		Produced by fibroblasts, neutrophils, and macrophages. Chemo-attractant for phagocytes at site of inflammation.

*The numbers in parentheses indicate the relevant references*.

**Figure 1:**
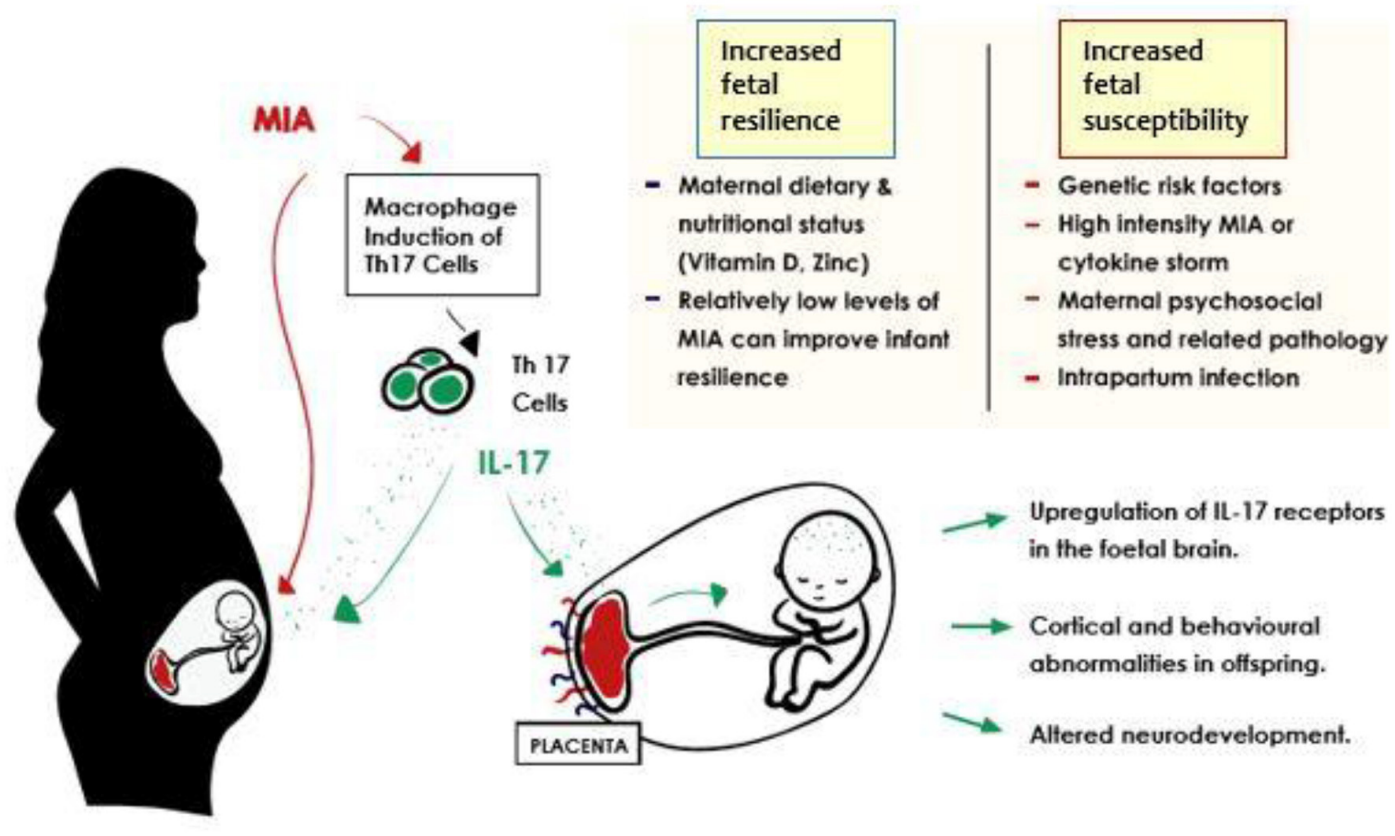
Potential outcomes in the inflammation-exposed fetus in the context of MIA related IL-17 induction. Improved fetal resilience is associated with lower intensity of maternal immune activation. Autism spectrum disorder risk after prenatal exposure to maternal fever has been found to increase in a dose dependent manner (55, 56) and similar effects were identified in animal models of MIA (57). A balanced maternal diet seems to contribute to improved fetal resilience also (58–60). Exposure to relatively higher grades of immune activation *via* high intensity MIA (40), intrapartum infection (61, 62), and genetic risk factors lead to reduced fetal resilience, and increased likelihood of unfavorable developmental outcomes.

A growing body of evidence supports a role in ASD pathogenesis for Th17 cells and their product cytokine, IL-17A (Figure 1) (79, 82). The IL17A gene itself has been identified by a small genome-wide CNV study to have amplified CNVs in ASD affected cohorts (83). Elevated levels of IL-17A have been reported in the blood of ASD affected individuals, and these correlate positively with severity of ASD behavioral symptoms (35, 63, 79). Yet, others have found high concentrations of IL-17A in individuals affected by obesity or high BMI (84), both of which are more likely in ASD groups (85). This is a potential confounder for any retrospective cohort based study designs.

**Figure 2:**
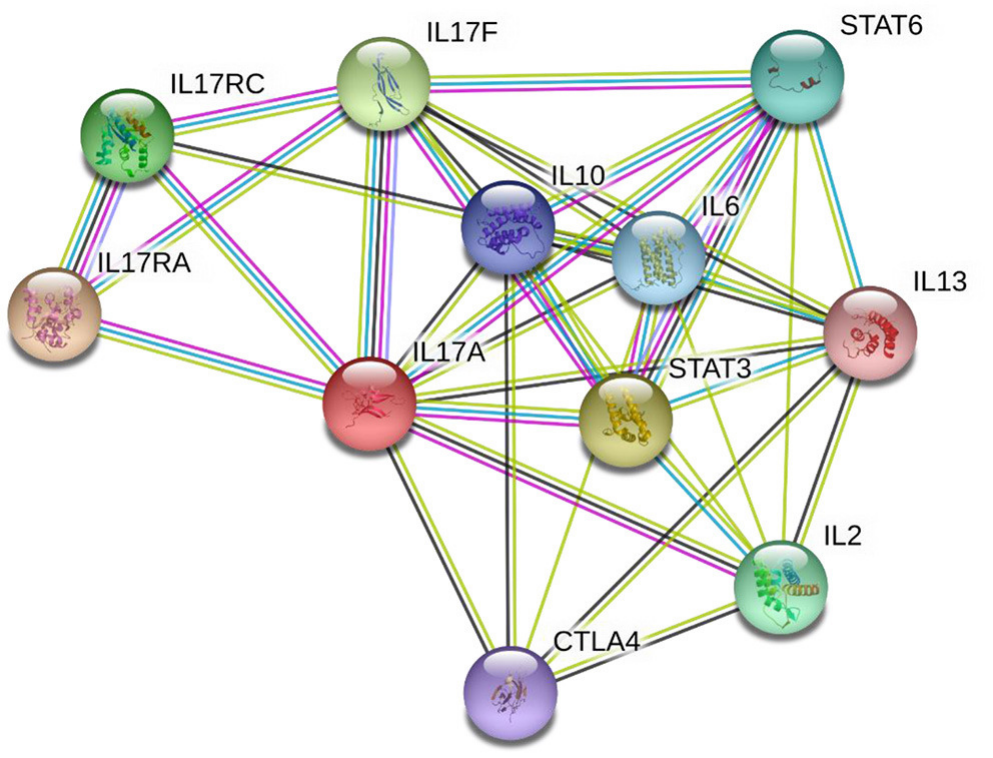
STRING diagram illustrating the known and predicted protein interactions for IL-17A. Network nodes represent proteins—each node represents all the proteins produced by a single, protein-coding gene locus. Edges (lines) represent protein-protein associations that are specific and meaningful, i.e., proteins jointly contribute to a shared function; this does not necessarily mean they are physically binding each other. Blue connecting lines indicate that protein interaction information was derived from curated databases, pink indicates the interaction was experimentally determined, yellow indicates the interaction was determined *via* text mining, black indicates protein co-expression, and lilac indicates protein homology. Analysis was performed on 28 July 2021 *via* the string-db.org domain.

STRING analysis (Figure 2) (86) indicates that IL-17A has proven or predicted interactions with IL-2, IL-6, IL-10, IL-13, IL-17F, IL-17RA, IL-17RC, CTLA4, STAT3, and STAT6. Each of these proteins have been previously reported to have altered expression in children with ASD, as outlined below. Of these, the most persistently described, and hence, potential key player is IL-17A, along with its receptor IL17RA and receptor complex, IL17RC.

Network nodes represent proteins—each node represents all the proteins produced by a single, protein-coding gene locus. Edges (lines) represent protein-protein associations that are specific and meaningful, i.e., proteins jointly contribute to a shared function; this does not necessarily mean they are physically binding each other. Blue connecting lines indicate that protein interaction information was derived from curated databases, pink indicates the interaction was experimentally determined, yellow indicates the interaction was determined *via* text mining, black indicates protein co-expression, and lilac indicates protein homology. Analysis was performed on 28 July 2021 *via* the string-db.org domain.

## IL-17A Associated pro-Inflammatory Mediators in ASD

Upregulation of pro-inflammatory pathways has been persistently associated with ASD. IL-6 is a versatile cytokine, with multiple functions throughout the body. It plays roles in immunity, inflammation, hematopoiesis, and oncogenesis. IL-6 works to promote pro-inflammatory Th17 cells (IL-17 producers) and to downregulate anti-inflammatory Treg cells (regulatory T-Helper cells) (87, 88). Th17 cells produce cytokines that cross the placental barrier (20). This transplacental effect has been well-characterized with IL-6, which was shown to alter offspring behavior and brain development (20, 89).

Like IL-17A, IL-17F is also produced by Th17 cells (90). IL-17F is reported to be involved in the regulation of proinflammatory gene expression and responses (91). IL-17RA and IL-17RC are both members of the IL-17 receptor family. In order for IL-17A (or indeed IL-17F) to have biological effects on tissues, IL-17RA must be present (90). IL-17RA is expressed in immune cells, and some children affected by ASD appear to possess higher levels of this receptor compared to neuro-typical controls (92). IL-17RA blockade may reduce monocyte associated oxidative stress which may improve neuro-inflammation associated with ASD (92). IL-17RC is also essential for the formation of the IL-17 receptor complex (46). IL-17RC levels in neutrophils are raised in children with ASD compared to neuro-typical controls. In fact, expression of this receptor (mRNA and protein) was completely absent in a cohort of neuro-typical children. The presence of both IL-17A receptor subunits in ASD patients may magnify the effects of IL-17A resulting in an autistic phenotype (93).

The transcription factor STAT3 (signal transducer and activator of transcription 3) is a key player in the development of T helper cells and regulates the expression of the T helper cell specific transcriptional regulator—retinoic acid receptor related orphan receptor γ-t (RORγt) *via* IL-6 (94, 95). IL-6 is a potent driver of RORγt activity. RORγt is exclusively found in lymphoid cells such as Th17 cells (CD 4 helper cells), and is required for differentiation of Tregs to Th17 cells (95). STAT3 proteins occur at elevated levels in the peripheral blood mononuclear cells (PBMCs) of children affected by ASD (96). Inhibition of STAT3 mitigates MIA associated behavioral and immunological abnormalities seen in animal models (49), while RORγt KO models reverse outcomes in MIA exposed mouse pups (18).

Lastly, IL-13 is a cytokine derived from T cells, which has both inflammatory and anti-inflammatory properties. IL-13 inhibits the production of other inflammatory cytokines (IL-1α, IL-1β, IL-6) through its effects on inflammatory macrophages (97). IL-13 is recognized as a key driver in allergic and inflammatory airway disease, where its effects are potentiated by IL-17 (98). Raised IL-13 has been noted in the plasma and PMBCs of children affected by ASD (29, 99), particularly those with comorbid asthma (although IL-13 is known to be skewed in those with co-morbid atopic conditions) (35).

## IL-17A Associated Anti-Inflammatory Mediators in ASD

Another member of the STAT family, STAT6, suppresses the IL-17A inflammatory response. In certain conditions, STAT6 signaling attenuates IL-17A producing T-cells, reducing their production of IL-17A (100). IL-4 mediated inhibition of Th17 cells and IL-17A production is STAT6 dependent (101). In human studies, children with ASD reportedly have reduced levels of STAT6-expressing CD45 cells (CD45^+^STAT6^+^) in their PBMC profile compared to neuro-typical controls (80). STAT6, as part of the IL-4 signaling cascade can enhance the expression of anti-inflammatory mediators. This pathway is critical for acceptance of the fetal graft, through reduction of Th17 cells and increase of both IL-4 and Tregs in the fetal environment (102, 103).

In addition to downregulation of the STAT6 mediated pathways, downregulation of other anti-inflammatory cytokines is also reported in autism. Anti-inflammatory cytokine IL-10 acts as a “master” immuno-regulator (104) and IL-10 concentrations are significantly lower in ASD children compared with neuro-typical controls (79, 105). Cytotoxic T-lymphocyte antigen 4 (CTLA4) is a glycoprotein located on T cells (106) and is induced following T cell activation. This anti-inflammatory molecule is expressed at lower levels in the PBMCs of children with ASD (107). Reductions in the levels of these anti-inflammatory and regulatory proteins may lead those with ASD to acquire a more pro-inflammatory state.

## Linking Immunity and Genetics in ASD

Bioinformatics analysis of large CNV studies suggest strongly that innate immune processes are implicated in ASD risk (108), this may indicate that immune dysfunction in ASD may be genetically driven or influenced. Maternal immune activation downregulates expression of susceptibility genes known to be highly penetrant in ASD and heavily involved in neurogenesis, cell signaling, synaptogenesis, and axonal guidance in the early stages of fetal development (108, 109). When compared with curated ASD associated gene sets [e.g., *via* the SFARI Gene database (http://gene.sfari.org/)], MIA downregulated genes were substantially enriched. The strongest enrichment of MIA downregulated genes was observed in the ASD gene categories with the highest likelihood of a link to ASD i.e., SFARI “High Confidence” or “Syndromic” ASD gene sets. This suggests that MIA may bestow increased ASD risk through downregulating the expression of the same genes that are highly penetrant in ASD during the early stages of fetal development.

Loss of function mutations in TSC1 and TSC2 genes are linked to syndromic ASD, and these genes are critical upstream regulators of the mammalian target of rapamycin (mTor) pathway. mTor has important functions in innate immunity and metabolism in particular (52, 110, 111).

Maternal immune activation also has downstream effects, in some cases influencing the transcriptome rather the genes themselves. Fragile X mental retardation 1 gene (FMR1) and CHD8 are both highly penetrant genes for ASD, yet MIA does not seem to influence expression of these genes directly. Rather, it wields an influence on downstream gene targets such as FMRP (fragile X syndrome protein complex). This raises the possibility that MIA may act as an environmental factor disrupting crucial early developmental genomic pathways through influence on downstream gene targets (108). This might suggest that MIA could act both in a direct (genetic) and indirect fashion (epigenetic/regulatory) with the end effects converging on similar pathways.

As previously, mentioned, normal pregnancy is associated with suppression of immunity, allowing the fetus to develop inside the mother's innate immune system. Human leukocyte antigen G coding gene antigen recognition controls the placental immune response and allows acceptance of the fetal graft. Human leukocyte antigen G coding gene interacts with the CD8 cell surface antigen found on most cytotoxic T-lymphocytes that mediate efficient cell–cell interactions within the immune system (112). Higher rates of HLA-G mutations have been found in mothers of children with ASD (113). The Th17 pathway in particular has been identified as a likely effector of inflammatory changes on the developing fetal brain, with downstream effects on behavior and cognitive development (46, 114). We hypothesize that the physiological changes in maternal immunity during pregnancy are dysregulated in some mothers of children with ASD.

In summary, many of the inflammatory proteins reported to have altered expression in ASD are linked to pro-inflammatory Th-17 cells, their product IL-17A, and the IL-17 receptors and receptor complexes. It appears that IL-6 activation (regulated by STAT3 and STAT6 *via* RORγt activity) of IL-17 expression, and subsequent upregulation of IL-17 receptors and receptor complexes may have a key role in the pathogenesis of ASD. The majority of linked molecules identified above are pro-inflammatory and found in higher quantities in those with ASD, with a corresponding downregulation of anti-inflammatory proteins. Whether this dysregulation of IL-17 is an inherent or acquired state is unclear.

Circulating T cell and IL-17A levels are altered in a subset of children with ASD. Maternal immune activation (including IL-17A) seems to play a role in altering important developmental pathways through direct interaction with ASD susceptibility genes, and indirectly, through interaction with their gene products. Circulating levels of IL-17A are dysregulated during pregnancy in mothers of children who develop ASD and ID (63, 79, 83). Murine models support a causative role for IL-17A in the pathogenesis of ASD. We conclude from the existing evidence that IL-17A dysregulation in the mother or developing infant could play a causal role in the development of at least some subsets of ASD and may be the link between environmental exposure and genetic susceptibility. Understanding the role of IL-17A and its associated targets on neurodevelopmental outcomes is now becomin increasingly important.

## What Is the Relevance of the Ongoing COVID-19 Pandemic to MIA-Induced ASD Risk?

Coronavirus disease 2019 (COVID-19), a disease caused by the novel coronavirus, SARS-CoV-2, has become a pandemic, affecting every corner of the globe. Although, the disease (COVID-19) affects primarily the respiratory systems of those affected, it has been found to affect and damage other organs, including the kidneys (115), liver (116), brain (117, 118), and heart (119, 120). Worldwide reported cases and COVID-19 related mortality are most likely an underestimate due to variability of public health capacities between countries, but as of August 2021, there have been almost 200 million confirmed cases of COVID-19, and over 4.2 million deaths reported to the WHO (121).

Our current knowledge of COVID-19 is based only on our limited experience with SARS-CoV-2 since December 2019 and analogously, through our experience of other coronaviruses (SARS CoV and MERS, Middle East Respiratory Syndrome). The long-term consequences of *in-utero* SARS-CoV-2 exposure and/or congenital infection are almost entirely unknown. There is clear evidence that prenatal exposure to viral infections increases the risk of adverse developmental, neurological, and psychiatric outcomes in later childhood and adult life (38, 44, 122). In this next section, we discuss the implications of the COVID-19 pandemic in the context of MIA-induced alterations in neurodevelopmental outcomes.

## COVID-19 and Cytokine Storm

Preclinical work shows that MIA, which stimulates interleukin-17A release from Th17 cells, can establish sustained fetal-placental inflammatory responses. This inflammatory milieu can persist into childhood and affect the development of the young “primed” brain. Remarkably, in murine models, social difficulties in MIA-exposed offspring are remediable through a variety of mechanisms including IL-17 blockade (18, 46). Cytokine storm is a general term applied to maladaptive cytokine release in responses to infection and other stimuli (123). In the context of sepsis, cytokine storm is considered one of the major causes of acute respiratory distress syndrome (ARDS), systemic inflammatory response syndrome (SIRS), and multi-organ failure (124, 125). In COVID-19, cytokine storm seems to play a role in disease aggravation and correlates positively with severity of disease (126). IL-17A target IL-6 and C-reactive protein (CRP) specifically, have been shown to correlate positively with increased mortality (127). Elevated numbers of Th17 cells have been isolated in the blood of individuals with fatal COVID-19 infection (128), while many authors have demonstrated significantly elevated levels of IL-17A in those with both mild and severe COVID-19 (129–131). Coronavirus infection results in macrophage, and dendritic cell activation and IL-6 release (132). This instigates an amplification cascade (JAK–STAT1/3 pathway) that results in cis signaling (binding of cell membrane bound IL-6 receptors) in lymphocytes with downregulation of Tregs and increased differentiation of TH17 cells; as well as trans-signaling (binding of soluble IL-6 receptor) effects on many other cell types (endothelial cells). This widespread immune activation and cytokine production contributes to the pathophysiology of severe COVID-19 (133). Indeed, some authors have specifically suggested therapies intended to target both Th17 cells and IL-17A in COVID-19 disease (134, 135). We have already outlined how Th17 specific (T-helper 17 cell) pathways are initiated *via* activated macrophages that produce IL-6 and IL-1β. As outlined, IL-6 in particular, is a potent potentiator and trigger for IL-17A release (123, 134, 136). IL-17A therefore, may be a key player in the COVID-19 cytokine storm.

## Coronavirus (SARS-CoV-2) Neurotropism and Neurological Effects

Coronaviruses have a demonstrated specific neuro-tropism that allows them access to, and to proliferate in, the host's CNS (137, 138). Cell entry seems to occur through the angiotensin-converting enzyme-2 (ACE-2) and transmembrane protease serine 2 (TMP S2) receptors, both of which are widely expressed in the placenta and at the feto-maternal interface. While trans-placental infection of the fetus is, yet to be proven conclusively, vertical transmission is certainly plausible and may lead directly to inflammatory processes in the fetal brain, in addition to indirect effects *via* the host/maternal immune response. The neurological sequelae of COVID-19 are wide-ranging and relatively common. The majority of neurological presentations so far have fallen into five categories, (i) Encephalopathy (including delirium and impaired consciousness), (ii) Inflammatory CNS disorders [including encephalitis and Acute Disseminating Encephalomyelitis (ADEM)], (iii) Cerebrovascular accident (CVA)/stroke, (iv) PNS disorders [including Guillain-Barré Syndrome (GBS) and cranial nerve palsies], (v) “Miscellaneous” central neurological disorders (such as raised intracranial pressure, seizures, and myelitis) (139). Hyposmia/Anosmia and hypogeusia (140) are recognized as two important hallmarks of acute SARS-CoV-2 infection, while more severe neurological complications have included CVAs, encephalitis, encephalopathy, and neuropsychiatric disorders (118, 141). Protein-protein network analysis for GBS and COVID-19 revealed that the combined gene set showed an increased connectivity as compared to COVID-19 or GBS alone, this was particularly true of genes related to Th17 cell differentiation. Transcriptome analysis of PBMC from patients with COVID-19 and GBS demonstrated the activation of interleukin-17 signaling in both conditions (142). Viral RNA has been isolated in clinical CSF samples in those with COVID-19 and neurological symptoms (143), and post-mortem examination of brain tissue has identified both viral RNA and neutrophilic infiltrates suggestive of aberrant immune response (144).

Recent pluripotent stem cell derived organoid models have been used to model SARS-CoV-2 infection in a wide range of tissues including gut, lung, liver, kidney, and brain (117, 145). These models demonstrate the virus' ability to infiltrate and proliferate in a variety of different cell/tissue types. Within the brain, the areas with the highest avidity for SARS-CoV-2 are the choroid plexus and the hippocampus (117). This is an interesting finding, as the choroid plexuses themselves represent the interface between CSFand blood compartments (in a similar fashion to the blood-brain barrier). They are located in each of the four ventricles, and are intimately related with immediately adjacent CSF, capillary blood supply, and neural tissue. Angiotensin-converting enzyme-2 receptors also appear to be highly expressed in the choroid plexus (146). In this sense, they provide a comprehensive roadmap upon which SARS-CoV-2 can potentially travel. The neurological features on COVID-19 infection are diverse and wide-ranging. Most studies to date have focused on symptomology in adult patients, but novel models of SARS-CoV-2 infection in a variety of human and animal tissues is casting new light on the mechanisms underlying COVID's infectivity and its ill-effects. There appears to be a variety of mechanisms underlying COVID's pathogenicity, not limited to direct viral effects on tissue, but also collateral effects *via* immune and thrombotic processes (147). Although there is little research on the effects of COVID on fetuses in early pregnancy, the same processes of direct viral effects and secondary immune and inflammatory effects are likely to be at play.

## Maternal COVID-19 Infection and Perinatal Exposure

Pregnant women are not thought to be more susceptible to contracting coronavirus than the general population (148), but given alterations in the pregnant immune state (103), they may be more susceptible to more severe infection (149, 150). Studies from previous pandemics, H1N1 influenza (2009), SARS (2003), and MERS (2012), suggest the possibility of significant maternal and neonatal morbidity and mortality (151, 152). Indeed, both MERS and SARS resulted in maternal death in a significant number of cases, but the specific risk factors for a fatal outcome during pregnancy are not clear. Our experience with these previous coronaviruses indicates higher risk of adverse outcomes for the fetus and infant including fetal growth restriction (FGR), and preterm delivery, both of which have previously been linked to increased ASD incidence (153) as well as NICU admission, spontaneous abortion, and perinatal death. As with other Coronaviruses, maternal SARS-CoV-2 infection has been associated with negative perinatal outcomes. Preterm delivery, fetal distress, stillbirth, and perinatal death have been widely reported (150, 154–156). Figures from China show that while up to 3% of pregnant women infected with COVID-19 required admission to intensive care (157, 158), a UK study showed 1% of pregnant women admitted with SARS-CoV-2 required ECMO (Extra-corporeal membrane oxygenation) and 10% Intensive Care Unit (ICU) management (159).

Cesarean section (CS) has been implicated as a risk factor for the development of ASD in offspring. The mechanisms underlying this are unclear, yet the risk of ASD is increased by approximately 33% in both elective and emergency CS procedures (160). In a systematic review of perinatal and maternal outcomes during the pandemic, CS rates were reported at extremely high levels, up to 90% in some centers (range from approximately 50–90%) (161). For comparison in work published in 2020, Turner at al noted an all-cause national CS rate in Ireland of approximately 26% (162). These higher rates were observed in most centers in spite of recommendations from the Royal College of Obstetrics and Gynecology (RCOG) and the International Federation of Gynecology and Obstetrics (IFGO) against decisions for CS being influenced by maternal SARS-CoV-2 status.

More specifically to neonatal outcomes, the WHO quotes worldwide preterm delivery rates of approximately 10% (163). Two large review studies reported preterm delivery rates of 20–25% in SARS-CoV-2 affected pregnancies (164, 165). Women with SARS-CoV-2 seemed to be more likely to endure a preterm delivery (165). The majority of these deliveries were iatrogenic, but in some reviews, up to half were attributable to either fetal or maternal compromise (166).

Maternal and neonatal ICU admission rates were also higher in the SARS-CoV-2 affected cohorts. Maternal ICU admission and mechanical ventilation rates were high vs. age matched non-pregnant women (165). While rates of stillbirth and neonatal death appear similar to uninfected fetuses, NICU admission rates were notably higher in COVID affected pregnancies (159), commonly as a precautionary step in the care of the neonate. Neonatal morbidity was higher in the SARS-CoV-2 affected groups and was associated with preterm delivery in mothers with more severe COVID-19 primary infection. Hypoxemia and respiratory difficulties in mothers had knock on effects of reduced placenta perfusion, pre-placental hypoxemia, fetal distress, and preterm delivery (167).

Given our knowledge of the potential developmental effects of Th17 activation in pregnancy, children *in-utero* during this pandemic may have significant inflammatory exposures if maternal infection occurs. There remain unanswered questions about the impact that asymptomatic and mild maternal infection has on the fetal brain in early pregnancy. Prospective follow up studies will need to follow infants whose mothers were infected as well as health unaffected controls. There is enormous potential to leverage archived serological samples from pregnancy and neonatal cohorts to study the relationships (or associations) between markers of maternal inflammation and later neurodevelopmental outcomes in offspring born during the pandemic. While in general, the likelihood of intrauterine maternal-fetal transmission of coronaviruses is low—there have been no documented cases of vertical transmission occurring with either SARS or MERS. There are current reports of possible vertical transmission of SARS-CoV-2 in several cases of third trimester maternal infection (168–170). Little to no information exists about children exposed in the first and second trimesters yet. While generally placental seeding does not seem common, some cases have reported strong evidence of placental infection with the demonstration of high viral load and immuno-histological evidence of SARS-CoV-2 in placental tissue (168). Currently, we can only surmise what the true effect (if any) of gestational COVID-19 on the incidence of ASD will be, but already some have concerns that the incidence may increase (171, 172). No studies have yet been reported on neurodevelopmental outcomes, as the oldest offspring are still in early childhood. Still, the evidence we have outlined within this review from MIA studies examining IL-17A and its pathway members provides a strong basis to build upon our current hypothesis and ask the question; could COVID-19 induced MIA act *via* IL-17A signaling to increase the risk of ASD-like phenotypes in vulnerable offspring?

## Discussion: Improving Outcomes for ASD Affected Individuals and Families

We believe that in spite of the tragedy of the COVID-19 emergency, we are presented with a serendipitous opportunity to progress scientific knowledge regarding prenatal exposures and ASD risk. During the COVID-19 pandemic, we have witnessed a novel infection, affect an immunologically naïve population within an extremely well-defined period of exposure. COVID-19 is now a notifiable illness, and has been characterized and monitored more than any illness in history. Many countries have developed stringent mandatory testing protocols, and track and trace programmes. Within all this, exists an opportunity to study the longitudinal effects of this infection on offspring of those affected by gestational COVID. Further investigation of mid-gestational cytokine profiles (IL-17A in particular) and their potential for genetic interplay could be a crucial cog in the development of actionable and cost-effective improvements in the current models of ASD care. Identification of pathways of immune dysregulation during pregnancy could lead to the identification of a risk marker of ASD that could be characterized in broader ASD cohorts. This would facilitate the identification of a predictive marker of ASD allowing earlier dedicated ASD screening in at risk children. Coupled with these potential biochemical markers, known early clinical signs of ASD exist. Crystallization of the ASD diagnosis can be as early as 14 months old according to some authors, and there are clinically detectable signs of ASD from a younger age still (23, 173, 174). The first children born of this pandemic are now reaching their toddler years, and they may represent a group with increased risk of ASD or other developmental conditions. Taken together, a postulated early biochemical marker and established early clinical markers could allow targeted early ASD screening, which would lead to earlier intervention, and improved outcomes. Therapies instituted in this age group have the potential to significantly improve clinical outcomes in ASD affected children. The timing of therapy is important with the most dramatic symptomatic and developmental improvements in those detected at an earlier age of diagnosis (175, 176).

We believe that it is the obligation of the scientific community to glean what benefit we can from this pandemic. In spite of social distancing measures, systematic national “lockdowns,” and working from home, there has been unprecedented scientific collaboration to try to counter the scourge of COVID. This has led to some outstanding success, not least in the development of two highly effective mRNA vaccines. In order to facilitate international research, the development of an international gestational COVID-19 consortium and registry would be an important step in coordinating research activities and aims. Isolation of relevant clinical bio-samples and prospective identification of patients will have already begun in some centers, and should be facilitated by the public health infrastructures that have been built up around the pandemic. Multidisciplinary collaborative follow up programmes should be established to identify, assess, and treat children with potential negative post-COVID outcomes.

## Data Availability Statement

The original contributions presented in the study are included in the article/supplementary material, further inquiries can be directed to the corresponding authors.

## Author Contributions

MC wrote the manuscript, reviewed the literature, and synthesized the hypothesis. SC, LGi, and LGa commented on the manuscript at all stages. GO'K commented on the manuscript and aided with literature review. DM commented on the manuscript, helped to synthesize the hypothesis, review the literature, and was the key supervisor. All authors have read and approved the final manuscript.

## Funding

Funding was provided to MC by the National Children's Research Center (NCRC) (grant D/19/1), Our Lady's Children's Hospital, Crumlin, Dublin 12, Ireland.

## Conflict of Interest

The authors declare that the research was conducted in the absence of any commercial or financial relationships that could be construed as a potential conflict of interest.

## Publisher's Note

All claims expressed in this article are solely those of the authors and do not necessarily represent those of their affiliated organizations, or those of the publisher, the editors and the reviewers. Any product that may be evaluated in this article, or claim that may be made by its manufacturer, is not guaranteed or endorsed by the publisher.

## Abbreviations

ACE-2: angiotensin-converting enzyme-2
ADHD: Attention Deficit Hyperactivity Disorder
ARDS: acute respiratory distress syndrome
ASD: autism spectrum disorder
CS: cesarean section
CD8 cell, cluster of differentiation 8: cytotoxic T-lymphocytes
CHD8: chromodomain helicase DNA binding protein 8 gene
CNV: copy number variant
COVID-19: corona virus disease-2019
FMR1: fragile X mental retardation 1 gene
GWAS: genome-wide association study
HLA-G gene: human leukocyte antigen G coding gene
ID: intellectual disability
IL: interleukin
IL17A gene: interleukin 17A gene
LPS: lipopolysaccharide
MERS: Middle Eastern Respiratory Syndrome
MIA: maternal immune activation
mTor: mammalian target of rapamycin
Poly (I:C): polyinosinic:polycytidylic acid
PNS: peripheral nervous system
RORγt: retinoid-related orphan receptor gamma t
SARS-CoV-2: severe acute respiratory syndrome-coronavirus 2
SNV: single nucleotide variant
Th17: T helper 17 cell
TSC1/TSC 2: Tuberous sclerosis complex ½.
